# Particle Size Effect of Cyetpyrafen Formulation in the Pesticide Transmission Process and Its Impact on Biological Activity

**DOI:** 10.3390/molecules28217432

**Published:** 2023-11-05

**Authors:** Lu Yu, He Liu, Miao Yu, Qi Zhang, Jingyu Chou, Yuanhua Wu

**Affiliations:** 1 Plant Protection College, Shenyang Agricultural University, No. 120 Dongling Road, Shenyang 110866, China; yulu_19841017@126.com (L.Y.); 2023500024@syau.edu.cn (H.L.); 2022200150@stu.syau.edu.cn (M.Y.); 2 State Laboratory of the Discovery and Development of Novel Pesticide, Shenyang Sinochem Agrochemicals R&D Co., Ltd., No. 8 Shenliao East Road, Shenyang 110021, China

**Keywords:** high-throughput formulation technology, size effect, cyetpyrafen, pesticide formulation, efficacy

## Abstract

Cyetpyrafen is a compound that lacks inherent uptake and systemic translocation activity. If mites do not come into direct contact with the pesticide solution on leaves, the efficacy cannot be achieved. Controlling the particle size can potentially play a crucial role in the manifestation of efficacy. In this study, high-throughput formulation technology was used to systematically screen a large number of adjuvants to obtain cyetpyrafen formulations. The particle size of the active ingredient in the formulation was measured. By examining the dynamic light scattering and contact angle, we simulated the actual process of the efficacy transmission of cyetpyrafen formulations against *Tetranychus cinnabarinus*. Our results showed that the activity of cyetpyrafen increases as the particle size decreases, suggesting that reducing the particle size can enhance the coverage and deposition on crop leaves, and further improve the dispersion efficiency and enhance spreading capabilities. Furthermore, controlling the particle size at 160 nm resulted in an LC_50_ value of 0.2026, which is approximately double than that of the commercial product. As a novel pesticide for mites, our study presents the most effective cyetpyrafen formulation in practice. Our findings provide valuable insights into controlling other mite species that pose a threat to agricultural products.

## 1. Introduction

In recent years, the damage caused by plant-feeding mites has intensified, and the frequent occurrence of mite resistance to the currently used acaricides has exacerbated the problem. Cyetpyrafen is a highly effective pesticide to manage mites in China [1]. However, in the form of acaricidal compounds, increased doses of application have led to a high level of mite resistance to cyetpyrafen [2,3]. The application of the cyetpyrafen compound mainly relies on dispersion transfer and interface transfer; however, it lacks inherent uptake and systemic translocation activity for efficient use in practice. Most research has focused on developing new efficient acaricidal compounds with novel modes of action, which is time-consuming and costly [4]. Thus, exploring alternative strategies to enhance the efficacy of formulations in acaricidal compounds is highly warranted.

In most cases, pesticide formulations are formed via manual large-scale adjuvant combinations to meet the formulation’s usability. With the advancement of automation technology, especially the rapid development of robotics, high-throughput technology, as a new rapid development technique of the 21st century, has been widely applied to medicine screening [5]. However, due to factors such as the high cost of advanced equipment investment and the limitations of thinking patterns, high-throughput formulation technology is relatively uncommon in practice. Currently, many studies focus on using a single type of adjuvant or a class of adjuvants to modify the structure, thereby altering the particle size and surface tension [6]. Screening and regulating the proportions of adjuvant systems on a large scale require substantial manpower and resources, involving multiple manual screening steps, and result in obtaining only a limited number of formulations with different sizes within a relatively long research and development period [7,8]. Moreover, research on the relationships between changes in the particle size of non-systemic pesticides due to complex adjuvant systems and various influencing factors during the transmission process warrants further investigation.

Previous studies have indicated that the conventional spraying of acaricidal formulations may not achieve comprehensive coverage of leaf surfaces, leading to the aforementioned issues with the effectiveness of the pesticide [9]. The main objective of this study is to find the particle size key to maximize the efficacy of acaricidal compounds without systemic activity. We hypothesize that size effects can impact the uniformity of the formulation dilution, drift losses during the spraying process, and the amount of active ingredient reaching the leaves [10]. The transfer processes of these three aspects are indeed key factors that influence the efficacy of the cyetpyrafen compound.

We utilized HTPS (high-throughput preparation and screening), a fully automatic high-throughput agrochemical formula preparation and screening system, specifically designed for pesticide formulations. It is an advanced approach that enables the rapid screening and evaluation of a large number of different formulations simultaneously. This innovative technique utilizes automated processes to test various combinations of ingredients, optimizing the formulation development process and accelerating the identification of optimal formulations for specific applications. It revolutionizes the traditional formulation approach by significantly reducing the time, cost, and resources required for formulation experimentation, ultimately leading to an improved efficiency and efficacy in the development of new products.

The impact of the particle size effect on the dosage transmission process is evaluated and analyzed using the dynamic light scattering index TSI [11]. Additionally, the dynamic contact angle changes from some existing research are combined for a comprehensive analysis of the liquid dosage transmission process of acaricidal compounds without systemic activity [12]. The study identifies the optimal effective ingredient particle size range and distribution of cyetpyrafen formulations during the dispersion transmission and interface transmission processes. Furthermore, indoor potted plant experiments were conducted using different-sized cyetpyrafen formulations to investigate mite mortality rates [13]. The present study has not only validated the practical applicability, but also identified the optimal range of ingredient particle size for cyetpyrafen formulations in the most effective way.

Research on acaricidal pesticide formulations holds significant importance. By understanding the particle size of active ingredients and its impact on efficacy, researchers can develop more effective and targeted acaricides [14]. This, in turn, can lead to improved pest control measures, increased crop yields, and reduced economic losses caused by mite infestations. Furthermore, advancements in pesticide formulation research can contribute to sustainable agriculture practices by minimizing the use of harmful chemicals and optimizing the effectiveness of pest management strategies [15].

## 2. Results and Discussion

### 2.1. Influence of the Size Effect on the Uniformity of Diluted Formulation 

Combining the appearance evaluation obtained from LEA PolyView and the particle size data obtained from Helos Quiexl, typical formulations were selected, and their particle size data are shown in Table 1.

The initial step in the application of a cyetpyrafen formulation is the dispersion process, where the active ingredient is diluted with water to form the liquid formulation. In this study, a dynamic light scattering analyzer was used to analyze the uniformity of cyetpyrafen formulations with different particle sizes. The variation in backscattered light intensity and transmitted light intensity over time were analyzed, enabling the detection of particle motion phenomena with cyetpyrafen formulations with different particle sizes [16].

Turbiscan Lab was used to perform scans according to pre-set programs, displaying the spectral profiles of different scan times on the same graph for comparison. As the detection time extended, the backscattered light intensity of the three parts of the sample tube continuously changed to indicate the uniformity of the particle motion. The variation in the curve was used to determine the changes in the system [17].

Ultimately, the TSI of the sample was quantitatively calculated. This index was derived from the raw data measured using the instrument, obtained directly from the backscattered light and transmitted light signals, and could characterize the degree of non-uniformity of the cyetpyrafen formulation sample [18].

**Figure 1:**
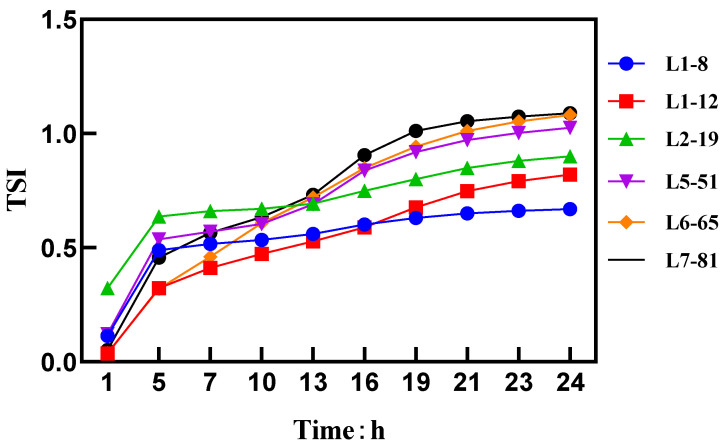
TSI values and curves.

A higher TSI value indicates a less uniform system. Figure 1 shows that as the particle size decreases, a significant decrease in the TSI was observed, indicating an improvement in the uniformity of the spray solution. The Brownian motion characteristics of the spray solution particles were measured using a dynamic light scattering instrument [19]. According to Stokes’ formula, the velocity of the particles undergoing Brownian motion was inversely proportional to their particle size.

**Figure 2:**
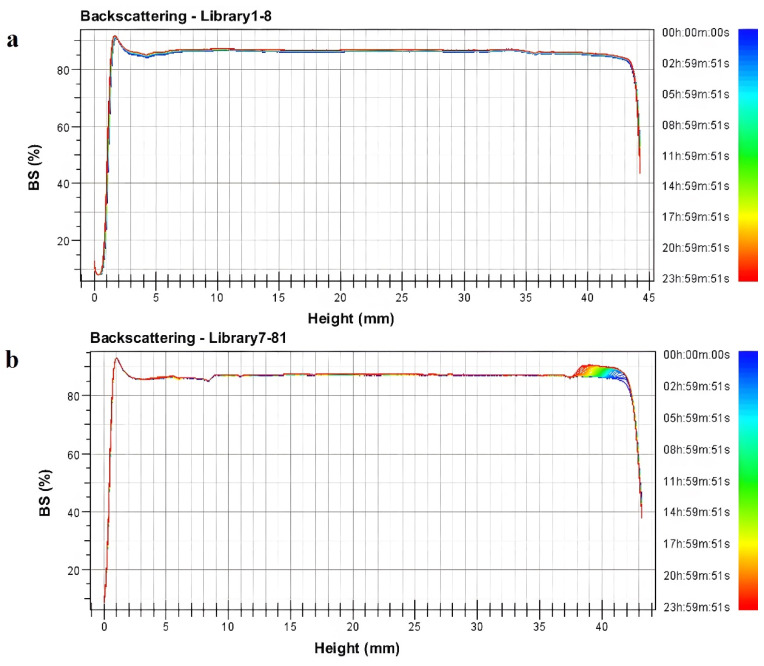
Backscattering light spectra (BS): (**a**) L1–8 sample; (**b**) L7–81 sample.

Further, combined with the transmitted and backscattered light spectra, an analysis of the non-uniformity phenomenon was conducted. Due to the opaqueness of the cyetpyrafen formulation, we used backscattered light for the analysis. The right side of the backscattered light fingerprint represents the top of the sample cell, while the left side represents the bottom. From Figure 2, it can be observed that the backscattered light intensity shows significant fluctuations on the right side of the spectra. Specifically, sample L1–8 exhibits a clear upward peak between 38 mm and 41 mm, indicating an increase in local particle concentration and the formation of particle aggregates. This suggests that the uniformity of L7–81 is noticeably weaker than that of L1–8, where the backscattered light intensity remains uniform within the same range [20].

The determination of the backscattered light was conducted using Turbiscan Lab with near-infrared light as the light source. With the variation in the sample incubation time, both the transmitted light and backscattered light underwent changes due to the sample’s instability. This indicates alterations in the particle size and concentration, thereby characterizing its instability.

There was a certain relationship between the particle size of the diluted formulation and Brownian motion. Smaller particle sizes (L1–8) exhibited a more pronounced irregular motion due to thermal agitation. These smaller particles had a higher surface area-to-mass ratio, resulting in more frequent collisions with surrounding molecules. Conversely, larger particle sizes (L7–81) had sufficient motion to counteract the effects of molecular collisions, resulting in a relatively uniform trajectory of motion [21].

### 2.2. Influence of the Size Effect on the Contact Angle 

An important process of cyetpyrafen formulation application involves interfacial transfer, specifically the process of droplets falling onto leaves, and adhering and permeating the leaf surface [22]. The contact angle is the most direct indicator of wetting and spreading properties. The contact angle hysteresis (advancing contact angle and receding contact angle) reflects the likelihood of a liquid sliding off the surface and is mainly determined based on the number of added surfactants in the pesticide solution, guiding the research on formulation enhancement [8]. In this study, by adding equal amounts of surfactants, we assumed and simulated that all droplets effectively fell on the leaves. We investigated the permeation and conduction ability of different-sized cyetpyrafen formulations on the leaf surface within 10 s for six typical sizes of cyetpyrafen formulations [23].

**Figure 3:**
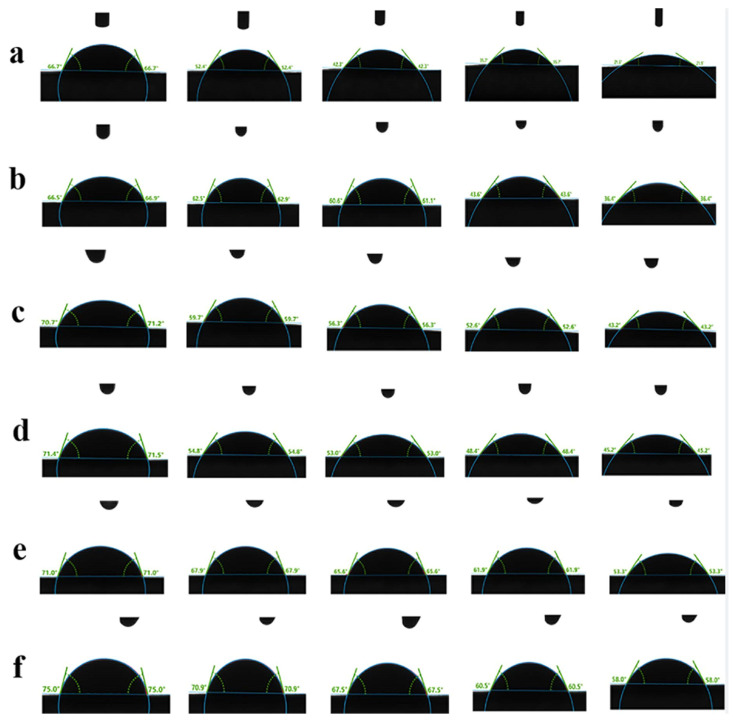
The wetting and spreading process within 10 s: (**a**) L1–8 sample; (**b**) L1–12 sample; (**c**) L2–19 sample; (**d**) L5–51 sample; (**e**) L6–65 sample; (**f**) L7–81 sample.

According to the analysis and calculations using advanced software, the dynamic changes in the wetting and spreading process, and contact angle within 10 s were obtained and are shown in Figure 3. To calculate the degree of change in the contact angle at a fixed time, the circle fitting method was used to calculate the contact angle. From Figure S1, it can be observed that with an increase in particle size, the magnitude of the change in the contact angle decreases. Among them, L1–8 (with a particle size of 160 nm) exhibited the steepest slope, indicating the largest decrease in the contact angle within 10 s and the strongest wetting and spreading ability. In contrast, for L7–81 (with a particle size of 15 μm), the needle position was no longer at the center of the droplet, indicating that the liquid had already started rolling on the leaf. This occurrence may lead to the possibility of the liquid rolling off the leaf during the actual application process, significantly affecting the efficacy [24].

### 2.3. Influence of the Size Effect on the Indoor Pot Efficacy and Deposition 

**Figure 4:**
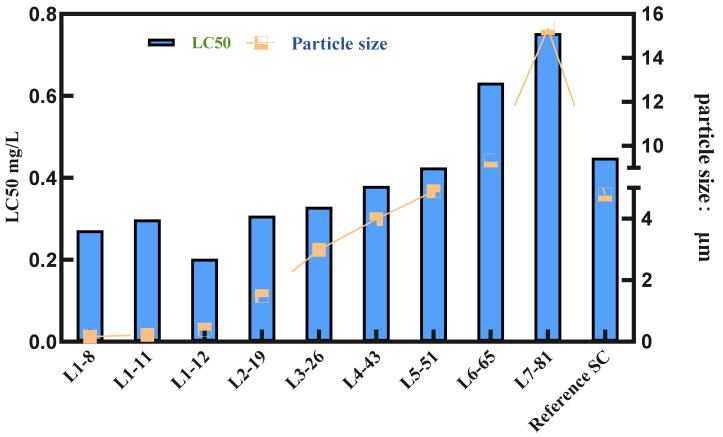
Relationship between the LC_50_ value and particle size.

To verify the parameters in the liquid transfer process and ensure the reliability of the selected formulations, we conducted pot seedling spray tests on nine samples and the commercial product cyetpyrafen 30% SC (the brand name and source of the reference formulation is a commercially available product, CORRE™) to evaluate their efficacy against *Tetranychus cinnabarinus*. The results are shown in Figure 4.

From Figure 4 and Table S1, it can be observed that the overall trend shows an increase in LC_50_ values and a gradual decrease in efficacy as the particle size of the cyetpyrafen formulation increases. Particularly, when the particle size exceeds 5 μm, there is a significant increase in LC_50_ values, indicating a notable decrease in efficacy. Considering indoor activity from a single factor, as the particle size decreases, the acaricidal activity of cyetpyrafen formulation gradually increases. When the particle size is 0.39 μm, the LC_50_ value is the lowest, indicating that its efficacy is twice as high as that of the current commercial product, cyetpyrafen SC.

**Figure 5:**
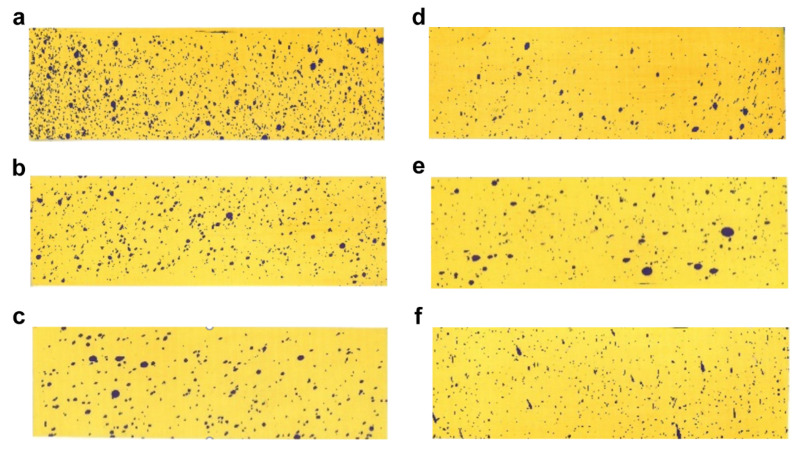
Deposition images: (**a**) L1–8; (**b**) L1–12; (**c**) L2–19; (**d**) L5–51; (**e**) L6–65; (**f**) L7–81.

To investigate the mechanism by which changes in formulation size affect the enhancement of efficacy, we selected six representative samples that have been characterized for uniformity and contact angle. By measuring the deposition and coverage of the spray after application on the six selected samples, we described the differences in the number of particles per unit area on target crops for different particle sizes. This allowed us to determine the varying impact of size changes on deposition and infer the pattern of efficacy changes [25]. As shown in Figure 5 and referring to Table S2, it is observed that the number of visible liquid droplets on the water-sensitive paper gradually decreased with the increase in particle size, leading to a corresponding decrease in the deposition amount. Among these, the L1–8 sample exhibited the highest number of visible liquid droplet depositions per unit area, with a deposition amount of 0.98 μL/cm^2^, significantly higher than the other samples.

The results of the spray deposition indicate that smaller particle sizes result in higher deposition amounts per unit area and increased coverage of formulation. This implies easier contact between the mites and the solution, thereby enhancing the efficacy. We can draw the conclusion that the reduction in particle size enhances the coverage of the pesticide on the target crop leaves.

### 2.4. Combining the Theoretical Transport Processes and Actual Efficacy 

We characterized the dynamic light scattering TSI and dynamic contact angle to simulate the dose transfer processes of dispersion and interfacial transfer in actual liquid transmission. The preliminary findings indicated the impact of the size effect on the efficacy as follows:

Cyetpyrafen is a non-systemic compound that acts upon contact with mites and exhibits activity against them. Its effectiveness is dependent on the mites coming into contact with the liquid formulation. Therefore, the more the liquid adheres to the leaves, the more pronounced the pesticidal effects will be [26].

Dispersion transfer: The uniformity of the diluted formulation is characterized using the dynamic light scattering theory. As the particle size decreases, there is a significant decrease in the TSI, indicating an improvement in the uniformity of the spray solution. This enhanced uniformity leads to improved dispersion efficiency, which in turn contributes to an enhancement in efficacy. When the particle size was smaller than 1.48 µm, both the TSI and backscattered light spectrum indicated better dispersion efficiency and improved uniformity of the sample. This was more advantageous for the efficacy to be exerted.

Interface transfer: Based on the results obtained from dynamic contact angle measurements, it is evident that the reduction in particle size significantly influences the wetting and spreading of a pesticide spray. Smaller particle sizes lead to an increased surface area, facilitating better wetting and spreading of the spray solution on target crops or surfaces. This enhanced wetting and spreading promote improved contact between the pesticide and the target organisms, thereby enhancing the effectiveness of the pesticide [27].

Considering the aforementioned conclusions, it can be preliminarily deduced that reducing the particle size offers potential benefits in improving efficacy. When considering the optimal values for indoor bioactivity assays, it was observed that a particle size of 160 nm for the cyetpyrafen formulation achieved the highest level of biological activity. Currently, when the particle size is further reduced beyond the scope of this study, it becomes challenging to obtain formulations on a large scale. In future work, further exploration of formulation techniques can be conducted to investigate the activity patterns of acaricides at even smaller sizes.

## 3. Materials and Methods

### 3.1. Materials

Cyetpyrafen (purity > 99%) and cyetpyrafen 30% SC were purchased from Shenyang Sciencreat Chemical Co., Ltd. (Shenyang, China). The specific adjuvants that fall under the industrial-grade category were directly purchased from manufacturers. The specific reference numbers for the different types and procurement source of adjuvants can be found in Table S3. The experimental organisms used were *Tetranychus cinnabarinus* (boisduval) and citrus plants.

### 3.2. Experimental Instruments

The fully automatic high-throughput agrochemical formula preparation and screening system (Freeslate, Unchained Labs of the USA, Pleasanton, CA, USA) was used in this study. LEA Library Studio, LEA Automation Studio, and LEA PolyView (all from Unchained Labs of the USA, Pleasanton, CA, USA) were also employed. The laser particle size distribution instrument (Helos Quiexl, SYMPATEC GmbH, Clausthal, Germany) was used for particle size analysis. The multi-sample stability analyzer (Turbiscan tower, Formulaction Inc., Toulouse, France) was utilized for uniformity analysis. A high shear emulsification machine (T18, IKA, Guangzhou, China) was used for emulsification. The contact angle measuring instrument (DSA100, Kruss GmbH, Hamburg, Germany) was employed to measure the contact angles. An electronic balance (XPR 205DU/AC, Mettler Toledo Technology (China) Co., Ltd., Shanghai, China) was used for weighing. The mist droplet test cards (measuring 7 cm in length and 3 cm in width) were purchased from the Institute of Plant Protection, Chinese Academy of Agricultural Sciences (Beijing, China). The scanning electron microscope (Model TS-215F) was purchased from Shanghai Zhongjing Technology Co., Ltd. (Shanghai, China). The mobile spray tower (Model 3WP-2000, equipped with TEEJET11004 nozzles) was obtained from the Nanjing Institute of Agricultural Mechanization, Ministry of Agriculture and Rural Affairs (Nanjing, China).

### 3.3. Screening Adjuvant Systems

#### 3.3.1. Unary Adjuvant Systems

HTPS was utilized to prepare the cyetpyrafen formulations. Ninety-six emulsifiers were screened using the LEA Library Studio, with input ratios of cyetpyrafen, solvent, adjuvant, and water, and 96 formulations were set up. The LEA Automation Studio was used to set up the operational procedure, placing all formulations in a 4 × 6 sample plate. The reaction vessels were 15 mL glass containers, and the specific procedure involved grabbing the provided ratios, weighing cyetpyrafen, returning the reaction vessels with robotic arms, and extracting the corresponding solvents from the reaction dishes. Each component was sequentially added to the reactors according to the program designed in LEA Library Studio, and magnetic stirring was carried out at 30 rpm for 10 min. The robotic arm’s suction head then extracted the emulsifiers from the reaction dishes, and each component was added to the reactors one by one, followed by magnetic stirring at 30 rpm for 20 min. The robotic arm individually picked up the uniformly mixed sample bottles and transferred them to the high-shear equipment position. Water was added to the sample bottles, and high-shear operations were performed at 12,000 rpm for 10 min, as per the previously set program. The HTPS equipment automatically took photographs to complete the preparation of the cyetpyrafen formulations.

**Figure 6:**
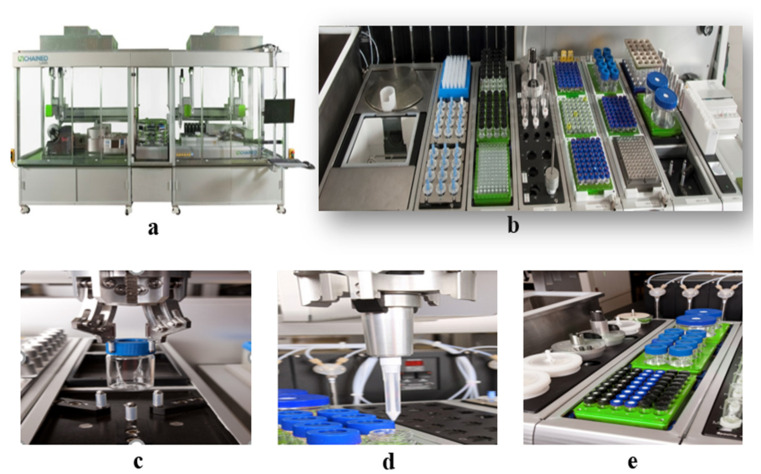
Construction of the high-throughput preparation and screening device. (**a**) appearance; (**b**) overall functional zone; (**c**) grab samples; (**d**) liquid measurement; (**e**) mixing process.

The construction of the HTPS is shown in Figure 6. The equipment includes the following: (a) appearance; (b-1) balance w/side-view camera; (b-2) PDT Rack and waste bin; (b-3) Vortexer mixing module; (b-4) combination hopper and tool rack for holding Powdernium™ Classic and SV hoppers and Dispense Tools for the Multifunctional Arm Element; (b-5) single-cooled position heated/tumbled stir bays; (b-6) capping station; (b-7) high-shear emulsification process; (c) grab samples; (d) liquid measurement; and (e) mixing process.

**Figure 7:**
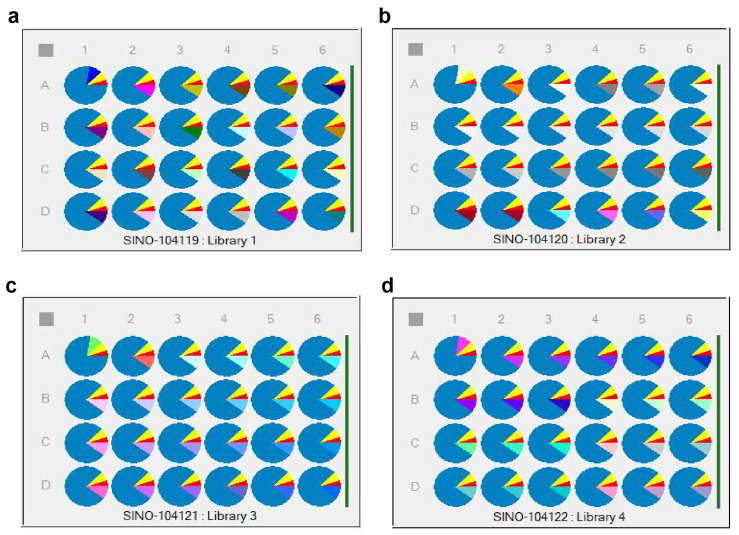
Distribution of the adjuvant systems in HTPS: 96 unary adjuvant system plates. (**a**) unary library 1 plate; (**b**) unary library 2 plate. (**c**) unary library 3 plate; (**d**) unary library 4 plate.

The first component was set as 5% cyetpyrafen (in red), the second component as 7.5% solvent S200 (in yellow), the third component as 10% of 96 different adjuvants (in various colors), and the fourth component as 77.5% water (in blue) [28]. The different unary adjuvant systems were divided into 4 × 6 samples per plate, with a total of 4 plates (Library 1~Library 4), as shown in Figure 7. Specific variables of the parts in various colors are listed in Tables S4–S7.

#### 3.3.2. Ternary Adjuvant Systems

Superior surfactants were selected from the unary surfactants with particle sizes below 20 µm. These selected adjuvant types are highlighted in bold in Tables S3–S6. These surfactants were arranged in ternary combinations, and following the same method as previously mentioned, all formulations were set up on a 3 × 4 sample plate. The HTPS operations and program settings are shown in Figure S2.

The selected unary adjuvants were further combined in ternary formulations to reduce the particle size and obtain different-size distributions. A total of 84 different combinations of cyetpyrafen formulations were obtained.

In the LEA Library Studio, the cyetpyrafen formulation was set up with the first component as 5% cyetpyrafen (in red), the second component as 7.5% solvent S200 (in green), the third to fifth components as 5% each of the 84 different ternary adjuvants (in various colors), and the fourth component as 72.5% water (in pink). The 84 different adjuvant systems were divided into 3 × 4 samples per plate, with a total of 7 plates (Library 1–Library 7), as shown in Figure S3. Specific variables of the parts in the other three colors are listed in Tables S8–S14.

### 3.4. Performance Measurement

#### 3.4.1. Effective Ingredient Particle Size Characterization

**Figure 8:**
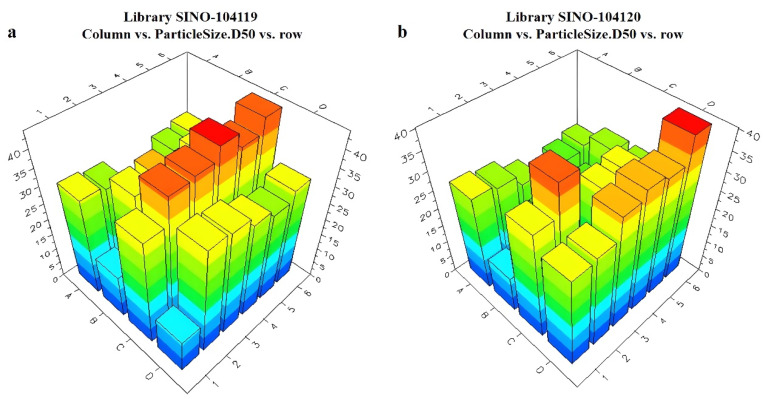
Relationship between different unary and ternary adjuvant systems and particle sizes: (**a**) unary library1 plate; (**b**) unary library 2 plate.

**Figure 9:**
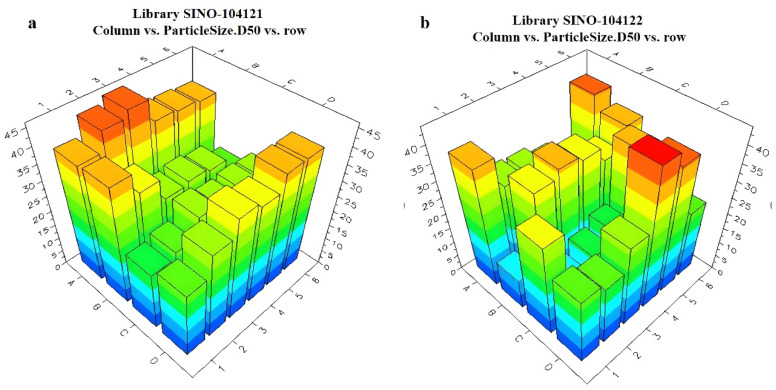
Relationship between different unary and ternary adjuvant systems and particle sizes: (**a**) unary library 3 plate; (**b**) unary library 4 plate.

The Helos Quiexl automatic testing program was set up to extractly 1 mL of the prepared pesticide liquid from the HTPS. It was then diluted with water to a total volume of 20 mL. The high-throughput particle size analyzer probe was sequentially inserted into each diluted sample bottle. When the light obscuration remained at 10–20%, the testing process began. The particle size data were obtained using the analyzer software, and the data were sent back to the LEA Library Studio. By comparing different formulations, a comparative analysis of the particle sizes of all samples was performed. The measurement results of the unary are presented in Figure 8 and Figure 9. The ternary systems are presented in Figure S4. The appearance of the formulations was also evaluated using LEA PolyView, as seen in Figure S5.

#### 3.4.2. Dynamic Light Scattering Measurement

Of the 15 selected samples of different sizes, 6 representative samples (L1–8, L1–12, L2–19, L5–51, L6–65, L7–81), each consisting of 5 mL, were diluted with water to a total volume of 100 mL. Then, 20 mL of the diluted solution was transferred to the measurement sample pool. Near-infrared light (880 nm) was used to perform scans from the bottom (0 mm) to the middle (20 mm) and to the top (45 mm) of the measurement pool. Scans were conducted every 5 min for a total of 24 h at a constant temperature of 30 °C. The measurements provided the intensity of transmitted and backscattered light for each sample, and the Turbiscan Stability Index (TSI) was calculated based on these measurements [29].

#### 3.4.3. Dynamic Contact Angle Measurement

Six representative samples (L1–8, L1–12, L2–19, L5–51, L6–65, L7–81) of different sizes were selected from the fifteen samples, each consisting of 5 mL, and diluted with water to a total volume of 100 mL. The contact angles of the six different dilutions were measured on citrus leaves. Healthy citrus leaves with a flat thickness (3 cm × 0.5 cm) were chosen, adhered to glass slides, and laid flat on the workbench. The droplet injector needle was fixed at a certain height, and an automated droplet dispensing system controlled by software was used. The fixed needle dispensed droplets with a precision of 0.1 µL, and the droplets were directly applied to the leaf surface.

The advanced software was used to further analyze and calculate the values of the contact angles during the droplet wetting and spreading process, and the data of the contact angles within the first 10 s of contact between the droplet and the leaf were analyzed [30].

### 3.5. Indoor Bioactivity Assay and Deposition Rate Representation

Nine samples (L1–8, L1–11, L1–12, L2–19, L3–26, L4–43, L5–51, L6–65, L7–81) from the previously mentioned fifteen samples and commercial product cyetpyrafen 30% SC were selected, with one from each interval. Each sample was applied to the target *Tetranychus cinnabarinus* at concentrations of 2.0, 1.0, 0.5, 0.25, and 0.125 ppm [31].

The bioactivity of the 10 samples against *Tetranychus cinnabarinus* was determined using a foliar spray method on potted seedlings. First, adult *Tetranychus cinnabarinus* mites were placed on citrus leaves, and the stable mite population was counted as the initial count. Following the experimental design, the mites were sprayed in a sequence from low to high doses, with each plant receiving 2 mL of spray, and each treatment was repeated three times. A blank control was also included.

The treated specimens were kept in an observation room under specified conditions, and the number of dead and alive mites was periodically recorded to calculate the mortality rate. DPS software (DPS V 20.05) was used to calculate the toxic regression equation, LC_50_, and 95% confidence limits.

Six samples (L1–8, L1–12, L2–19, L5–51, L6–65, L7–81) were selected from a total of 10 groups. Six sets of cyetpyrafen solutions were prepared at a medium concentration of 0.5 ppm for spray deposition testing. The mist droplet test cards were placed at the bottom of the spray tower, with a running speed of 400 mm/s, running distance of 1300 mm, and application rate of 50 mL. After spraying, the mist droplet test cards were scanned using a scanning electron microscope to generate grayscale images. The water-sensitive paper on the surface of the test card changed from yellow to blue upon contact with water. The droplet density (DD), fraction coverage (FC), and droplet rate (DR) were determined using ImageJ DepositScan software (ImageJ 1.40).

## 4. Conclusions

We characterized the influencing factors in terms of efficacy expression and studied the final biological activity. The findings showed that the particle size of the active ingredient was correlated with the efficacy of cyetpyrafen. Furthermore, the activity of the cyetpyrafen formulation increases with decreasing particle size. The results of deposition indicate that smaller particle sizes result in higher deposition amounts per unit area and an increased coverage of the formulation. Additionally, reducing the particle size can improve dispersion efficiency and enhance spreading capabilities. Moreover, controlling the particle size at 160 nm yielded an LC_50_ value of 0.2026, approximately twice that of the commercially available product. This study established a new approach for considering particle size effects as a variable in determining efficacy across the consecutive processes of dispersion transfer and interfacial transfer. Simultaneously, this work explored the possibility of transitioning pesticide formulation research from a random and simplistic theoretical guidance approach to a systematic and intelligent research mode. This laid the foundation for a new model and platform in agricultural chemical formulation research based on big data. In general, reducing the particle size enhances the coverage of the pesticide on the target crop leaves and improves its penetration capability on the crop surface. According to Stokes’ law, when the particle size decreases, the solid particles in the pesticide solution disperse more uniformly, resulting in an increased coverage by the pesticide solution [32]. It is important to note that this study focuses on cyetpyrafen formulations as an example, using particle size control to address issues related to the optimal particle size range and the impact of size effects on rheological properties and dispersion stability, which could provide a theoretical basis for future formulation efficacy studies.

## Figures and Tables

**Table 1:** SAA components and the particle size of typical samples.

Sample	No.	SAA Components	Particle Size (μm)
Library1-B2	L1-8	SK-20TX/SK-560EP/500#	0.16
Library1-C3	L1-11	SK-20TX/YUS-CH7000/AEO-3	0.22
Library1-C4	L1-12	SK-20TX/YUS-CH7000/500#	0.39
Library2-A3	L2-15	SK-20TX/Greenmul 5810/Emulsogen EL360	0.92
Library2-A4	L2-16	SK-20TX/Greenmul 5810/AEO-3	0.69
Library2-B3	L2-19	SK-20TX/Atlas G-1086/Emulsogen EL360	1.48
Library2-C2	L2-22	SK-20TX/Atlas G-1086/600#	1.82
Library3-A2	L3-26	SK-20TX/AEO-3/500#	2.98
Library3-B2	L3-30	SK-560EP/YUS-CH7000/Atlas G-1086	2.11
Library4-B3	L4-43	SK-560EP/Atlas G-1086/600#	3.98
Library4-B4	L4-44	SK-560EP/Emulsogen EL360/AEO-3	3.27
Library5-A3	L5-51	YUS-CH7000/Greenmul 5810/Emulsogen EL360	4.89
Library6-B1	L6-65	Greenmul 5810/Atlas G-1086/AEO-3	9.33
Library6-C4	L6-72	Greenmul 5810/AEO-3/500#	12.66
Library7-C1	L7-81	Emulsogen EL360/AEO-3/500#	15.33

## Data Availability

Data are available within the article.

## Supplementary Materials

The following supporting information can be downloaded at: https://www.mdpi.com/article/10.3390/molecules28217432/s1, Figure S1: Contact angle value within 10 s; Figure S2: Ternary adjuvant systems of HTPS; Figure S3: Distribution of adjuvant systems in HTPS: (a–g) 84 ternary adjuvant systems plates; Figure S4: Relationship between different ternary adjuvant systems and particle sizes; Figure S5: Appearance of 84 cyetpyrafen formulation samples prepared using HTPS; Table S1:Efficacy against *Tetranychus cinnabarinus* of nine samples and the reference sample; Table S2: Deposition results; Table S3: List of the source of adjuvant; Tables S4–S7: Unitary adjuvant systems (library 1–4 plate); Tables S8–S14: Ternary adjuvant systems (library 1–7 plate).

## Abbreviations

LC_50_	Lethal concentration 50
TSI	Turbiscan stability index
SC	Suspension concentrates
HTPS	High-throughput preparation and screening
SAA	Surface active agent
L1-1	Library 1-1 (L2-1 Library 2-1…)
