# Supplementary material for: Particle Size Effect of Cyetpyrafen Formulation in the Pesticide Transmission Process and Its Impact on Biological Activity
Source: Molecules. 2023 Nov 5;28(21):7432. doi: 10.3390/molecules28217432 (PMC10648920; doi:10.3390/molecules28217432)
Supplement: Supplementary file 1 [file molecules-28-07432-s001.zip › molecules-2668282-supplementary.pdf]

Supplementary Material

Particle Size Effect of Cyetpyrafen Formulation in the Pesticide Transmission Process and Its Impact on Biological Activity

Lu Yu[1, 2], He Liu[1], Miao Yu[1], Qi Zhang [1, *], Jingyu Chou[2, *], Yuanhua Wu[1, *]

[1]*Plant Protection college, Shenyang Agricultural University, Shenyang 110866, China*

[2]*Shenyang Sinochem Agrochemicals R&D Co. Ltd, State Laboratory of the Discovery and Development of Novel Pesticide, Shenyang 110021, China*

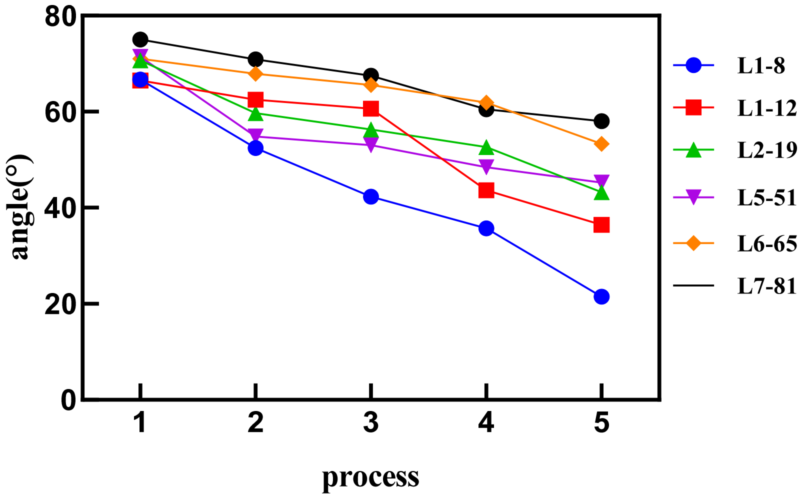

Figure S1. Contact angle value within 10 seconds

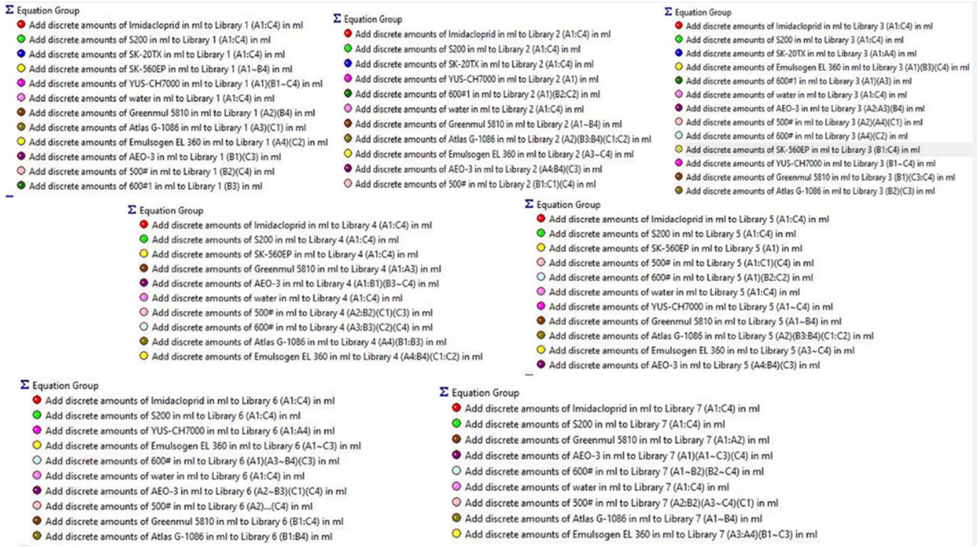

Figure S2. Ternary adjuvant systems of HTPS

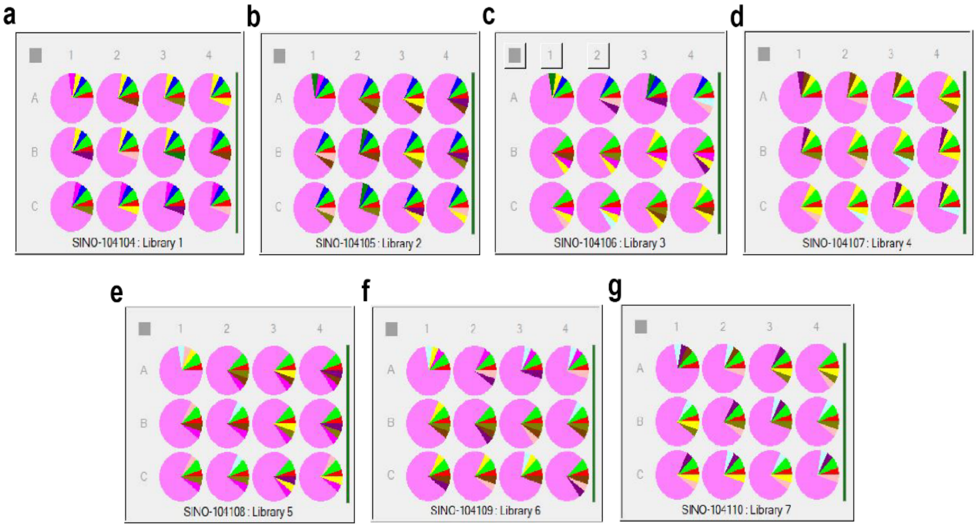

Figure S3. Distribution of adjuvant systems in HTPS: (a~g) 84 ternary adjuvant systems plates.

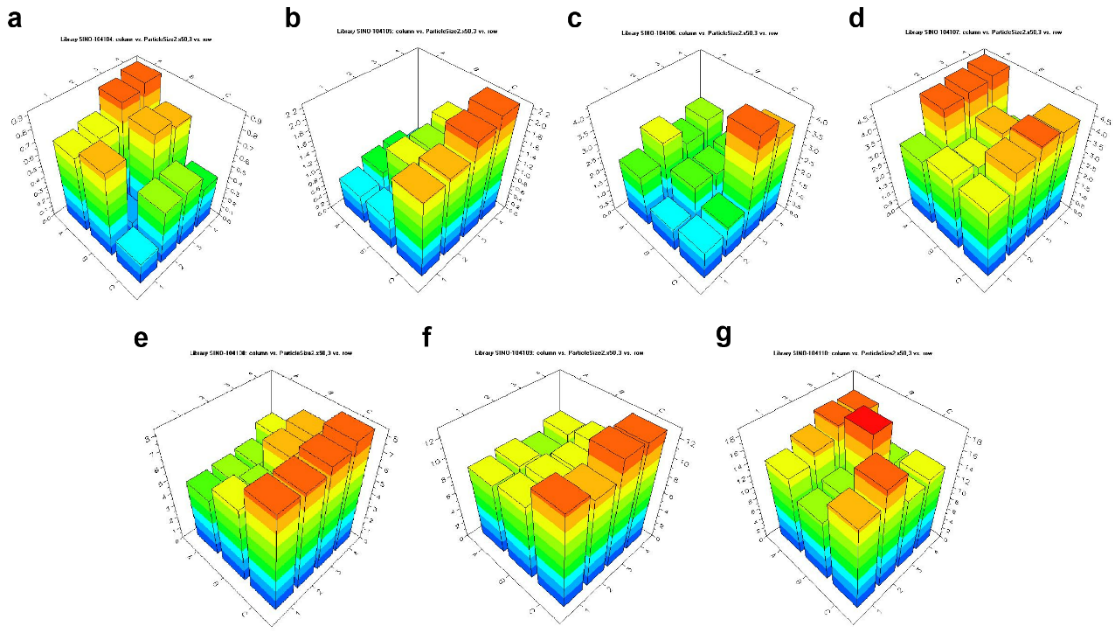

Figure S4. Relationship between different ternary adjuvant systems and particle size: (a) ternary library1 plate; (b) ternary library2 plate; (c) ternary library3 plate; (d) ternary library4 plate; (e) ternary library5 plate; (f) ternary library6 plate; (g) ternary library7 plate.

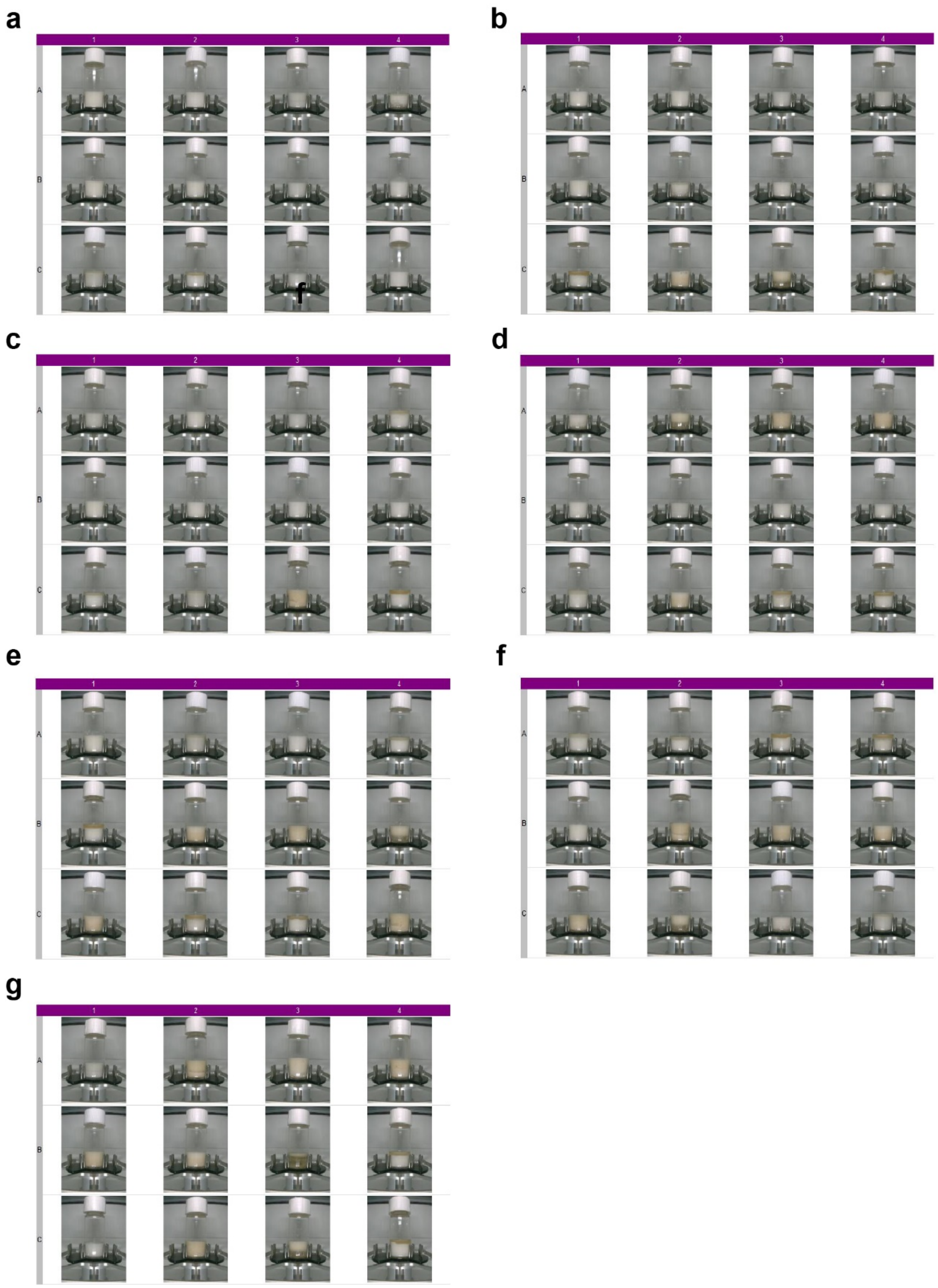

Figure S5. Appearance of 84 cyetpyrafen formulation samples prepared using HTPS: (a) appearance of samples from Library1-1 to Library1-12; (b) appearance of samples from Library2-1 to Library2-12; (c) appearance of samples from Library3-1 to Library3-12; (d) appearance of samples from Library4-1 to Library4-12; (e) appearance of samples from Library5-1 to Library5-12; (f) appearance of samples from Library6-1 to Library6-12; (g)appearance of samples from Library7-1 to Library7-12.

Table S1. The efficacy against the *Tetranychus cinnabarinus* of nine samples and reference sample.

Test sample	Particle size (μm)	Regression equation y=a+bx	LC50 (mg/L)	95% Confidence interval
Library 1-8	0.16	7.6414+4.6719x	0.2720	0.1663-0.3504
Library 1-11	0.22	8.0858+5.8738x	0.2983	0.1268-0.3978
Library 1-12	0.39	8.3149+4.7872x	0.2026	0.1554-0.2431
Library 2-19	1.48	7.4809+4.8428x	0.3074	0.2844-0.3715
Library 3-26	2.98	6.8834+3.0926x	0.3291	0.2872-0.4679
Library 4-43	3.98	6.6932+4.0315x	0.3802	0.3670-0.4886
Library 5-51	4.89	5.7861+2.1163x	0.4252	0.3935-0.5094
Library 6-65	9.33	5.5572+2.7971x	0.6321	0.4571-0.9604
Library 7-81	15.33	5.2143+1.7394x	0.7530	0.6304-0.9307
Reference SC	4.99	5.8373+2.4073x	0.4489	0.3935-0.5094

Table S2. Deposition results

	Fraction coverage (percentage)	Droplet density (per square centimeter)	Droplet rate (microliters per square centimeter)
**L1-8**	9.12±0.06a	37.75±0.25a	0.98±0.05a
**L1-12**	6.32±0.08b	34.93±0.50b	0.73±0.03b
**L2-19**	6.00±0.05bc	32.79±0.12b	0.52±0.06c
**L5-51**	5.64±0.12c	30.30±0.80c	0.51±0.03c
**L6-65**	4.26±0.09d	23.90±0.10d	0.44±0.02cd
**L7-81**	3.25±0.03e	20.47±0.73e	0.34±0.03d

The data analysis was conducted using SPSS 17.0 software. Univariate analysis of variance was employed to test for differences under the condition of a significance level of 95%.

Table S3. List of source of adjuvant

NO.	Adjuvant	Manufacturer	No.	Adjuvant	Manufacturer
1	SK-551	SK. AgroThink	49	Lissapol PA	Croda International Plc.
2	SK-560EP	(Shanghai) Corp.	50	Atlox4838B	
3	SK-273F1		51	Atlas G5000	
4	SK-5050		52	Cresplus DP	
5	SK-33SC		53	Cresmer SC947	
6	SK-01JK		54	Creslox 3484	
7	SK-34SC		55	Creslox 3418	
8	SK-20TX		56	Creslox 793	
9	SK-92FS1		57	3478N/803	
10	SK-24		58	Atlox 3416	
11	SK-21K		59	Creslox SC565	
12	SK-24R		60	Creslox SC802	
13	SK-25CH		61	Creslox SC671	

14	YUS-FS3000	Takemoto Oil & Fat Co., Ltd.	62	Arlatone TV	
15	YUS-WG4		63	Atlas G-1086	
16	YUS-207K		64	Atlas G5000	
17	YUS-TXC		65	Atlas G5002L	
18	YUS-RXB		66	Atlox 4896	
19	YUS-CH7000		67	Atlox 4894	
20	PICO-SC50Q		68	Atlox 4894A	
21	PICO-SC25P		69	Cresmer A5	
22	YUS-FS7PG		70	Synperonic 13/5	
23	YUS-FS1		71	Synperonic91/6	
24	YUS-SXC		72	Cresmer SC157	
25	YUS-LXC		73	Phenylsulfonat CA	Clariant International Ltd.
26	YUS-135B		74	Phenylsulfonat CAL	
27	YUS-2011CX		75	Emulsogen 3510	
28	YUS-2010CX		76	Emulsogen EL360	
29	YUS-D625		77	Emulsogen TS160	
30	YUS-D935		78	Emulsogen MP1	
31	YUS-D3020		79	SP27001	Jiangsu SinvoChem S&T Co. Ltd.
32	YUS-5050PB		80	SPOF3468	
33	YUS-CP120		81	SVA-116B	
34	YUS-EP70G		82	OD-3310	Dow Chemical (Shanghai) Co., Ltd.
35	YUS-A41B		83	W600	
36	YUS-110		84	D800/D865	
37	Greenmul 5038	Beijing Green-times Technology Co., Ltd.	85	TRITO GR-7M	
38	Greenmul 5800		86	DOWFA 2A1	
39	Greenmul 5810		87	AEO-3	Xingtai Yancheng Chemical Auxiliaries Co., Ltd.
40	Greenmul 5816		88	300#	
41	Greenmul 5860		89	500#	
42	Greenmul 8500		90	600#	
43	Greenmul 8600		91	700#	
44	Greenmul EF81		92	1600#	
45	Greenmul EF83		93	201	
46	TERMUL5030	Indorama Ventures Mobility Trading Co.,Ltd.	94	0201B	
47	TERMUL200		95	0203B	
48	TERMUL128x		96	EthylanNS-500LQ	Nanjing Jierun Sci. Tech. Co., Ltd

Table S4. Unitary adjuvant systems (Library 1 plate)

	**1**	**2**	**3**	**4**	**5**	**6**

A	SK-551	SK-5050	SK-01JK	**SK-20TX**	SK-24	SK-24R
B	**SK-560EP**	SK-33SC	SK-34SC	SK-92FS1	SK-21K	SK-25CH
C	SK-273F1	YUS-FS3000	YUS-WG4	YUS-207K	YUS-TXC	YUS-RXB
D	**YUS-CH7000**	PICO-SC50Q	PICO-SC25P	YUS-FS7PG	YUS-FS1	YUS-SXC

Table S5. Unitary adjuvant systems (Library 2 plate)

	**1**	**2**	**3**	**4**	**5**	**6**
A	YUS-LXC	TERMUL503 0	TERMUL200	TERMUL128 x	Greenmul 5038	Greenmul 5800
B	**Greenmul 5810**	Greenmul 5816	Greenmul 5860	Greenmul 8500	Greenmul 8600	Greenmul EF81
C	Greenmul EF83	Arlatone TV	**Atlas G-1086**	Atlas G5000	Atlas G5002L	Atlox 4896
D	Atlox 4894	Atlox 4894A	Cresmer A5	Synperonic 13/5	Synperonic91/6	Cresmer SC157

Table S6. Unitary adjuvant systems (Library 3 plate)

	**1**	**2**	**3**	**4**	**5**	**6**
A	Lissapol PA	Atlox4838B	Atlas G5000	Cresplus DP	Cresmer SC947	Creslox 3484
B	Creslox 3418	Creslox 793	3478N/803	Atlox 3416	YUS-135B	YUS-2011CX
C	YUS-2010CX	YUS-D625	YUS-D935	YUS-D3020	YUS-5050PB	YUS-CP120
D	YUS-EP70G	YUS-A41B	YUS-110	Ethylan NS-500LQ	Phenylsulfonat CA	Phenylsulfonat CAL

Table S7. Unitary adjuvant systems (Library 4 plate)

	**1**	**2**	**3**	**4**	**5**	**6**
A	Emulsogen 3510	**Emulsogen EL360**	Emulsogen TS160	Emulsogen MP1	SPOF3468	SVA-116B
B	**AEO-3**	OD-3310	W600	D800/D865	TRITO GR-7M	DOWFA 2A1
C	300#	**500#**	**600#**	700#	1600#	0201
D	0201B	0203B	Creslox SC565	Creslox SC802	Creslox SC671	SP27001

Table S8. Ternary adjuvant systems (Library 1 plate)

	**1**	**2**	**3**	**4**
**A**	SK-20TX<br>SK-560EP<br>YUS-CH7000	SK-20TX<br>SK-560EP<br>Greenmul 5810	SK-20TX<br>SK-560EP<br>Atlas G-1086	SK-20TX<br>SK-560EP<br>Emulsogen EL360
**B**	SK-20TX<br>SK-560EP<br>AEO-3	**SK-20TX**<br>**SK-560EP**<br>**500#**	SK-20TX<br>SK-560EP<br>600#	SK-20TX<br>YUS-CH7000<br>Greenmul 5810

C	SK-20TX<br>YUS-CH7000<br>Atlas G-1086	SK-20TX<br>YUS-CH7000<br>Emulsogen EL360	**SK-20TX**<br>**YUS-CH7000**<br>**AEO-3**	**SK-20TX**<br>**YUS-CH7000**<br>**500#**

Table S9. Ternary adjuvant systems (Library 2 plate)

	1	2	3	4
A	SK-20TX<br>YUS-CH7000<br>600#	SK-20TX<br>Greenmul 5810<br>Atlas G-1086	**SK-20TX**<br>**Greenmul 5810**<br>**Emulsogen EL360**	**SK-20TX**<br>**Greenmul 5810**<br>**AEO-3**
B	SK-20TX<br>Greenmul 5810<br>500#	SK-20TX<br>Greenmul 5810<br>600#	SK-20TX<br>Atlas G-1086<br>Emulsogen EL360	SK-20TX<br>Atlas G-1086<br>AEO-3
C	SK-20TX<br>Atlas G-1086<br>500#	**SK-20TX**<br>**Atlas G-1086**<br>**600#**	SK-20TX<br>Emulsogen EL360<br>AEO-3	SK-20TX<br>Emulsogen EL360<br>500#

Table S10. Ternary adjuvant systems (Library 3 plate)

	1	2	3	4
A	SK-20TX<br>Emulsogen EL360<br>600#	**SK-20TX**<br>**AEO-3**<br>**500#**	SK-20TX<br>AEO-3<br>600#	SK-20TX<br>500#<br>600#
B	SK-560EP<br>YUS-CH7000<br>Greenmul 5810	**SK-560EP**<br>**YUS-CH7000**<br>**Atlas G-1086**	SK-560EP<br>YUS-CH7000<br>Emulsogen EL360	SK-560EP<br>YUS-CH7000<br>AEO-3
C	SK-560EP<br>YUS-CH7000<br>500#	SK-560EP<br>YUS-CH7000<br>600#	SK-560EP<br>Greenmul 5810<br>Atlas G-1086	SK-560EP<br>Greenmul 5810<br>Emulsogen EL360

Table S11. Ternary adjuvant systems (Library 4 plate)

	1	2	3	4
A	SK-560EP<br>Greenmul 5810<br>AEO-3	SK-560EP<br>Greenmul 5810<br>500#	SK-560EP<br>Greenmul 5810<br>600#	SK-560EP<br>Atlas G-1086<br>Emulsogen EL360
B	SK-560EP<br>Atlas G-1086<br>AEO-3	SK-560EP<br>Atlas G-1086<br>500#	**SK-560EP**<br>**Atlas G-1086**<br>**600#**	**SK-560EP**<br>**Emulsogen EL360**<br>**AEO-3**
C	SK-560EP<br>Emulsogen EL360<br>500#	SK-560EP<br>Emulsogen EL360<br>600#	SK-560EP<br>AEO-3<br>500#	SK-560EP<br>AEO-3<br>600#

Table S12. Ternary adjuvant systems (Library 5 plate)

	1	2	3	4
A	SK-560EP	YUS-CH7000	**YUS-CH7000**	YUS-CH7000

	500#	Greenmul 5810	**Greenmul 5810**	Greenmul 5810
	600#	Atlas G-1086	**Emulsogen EL360**	AEO-3
	YUS-CH7000	YUS-CH7000	YUS-CH7000	YUS-CH7000
**B**	Greenmul 5810	Greenmul 5810	Atlas G-1086	Atlas G-1086
	500#	600#	Emulsogen EL360	AEO-3
	YUS-CH7000	YUS-CH7000	YUS-CH7000	YUS-CH7000
**C**	Atlas G-1086	Atlas G-1086	Emulsogen EL360	Emulsogen EL360
	500#	600#	AEO-3	500#

Table S13. Ternary adjuvant systems (Library 6 plate)

	**1**	**2**	**3**	**4**
	YUS-CH7000	YUS-CH7000	YUS-CH7000	YUS-CH7000
**A**	Emulsogen EL360	AEO-3	AEO-3	500#
	600#	500#	600#	600#
	Greenmul 5810	**Greenmul 5810**	Greenmul 5810	Greenmul 5810
**B**	Atlas G-1086	**Atlas G-1086**	Atlas G-1086	Atlas G-1086
	Emulsogen EL360	**AEO-3**	500#	600#
	Greenmul 5810	Greenmul 5810	Greenmul 5810	**Greenmul 5810**
**C**	Emulsogen EL360	Emulsogen EL360	Emulsogen EL360	**AEO-3**
	AEO-3	500#	600#	**500#**

Table S14. Ternary adjuvant systems (Library 7 plate)

	**1**	**2**	**3**	**4**
	Greenmul 5810	Greenmul 5810	Atlas G-1086	Atlas G-1086
**A**	AEO-3	500#	Emulsogen EL360	Emulsogen EL360
	600#	600#	AEO-3	500#
	Atlas G-1086	Atlas G-1086	Atlas G-1086	Atlas G-1086
**B**	Emulsogen EL360	AEO-3	AEO-3	500#
	600#	500#	600#	600#
	**Emulsogen EL360**	Emulsogen EL360	Emulsogen EL360	AEO-3
**C**	**AEO-3**	AEO-3	500#	500#
	**500#**	600#	600#	600#
